# A descriptive systematic review of the relationship between personality traits and quality of life of women with non-metastatic breast cancer

**DOI:** 10.1186/s12885-022-09408-4

**Published:** 2022-04-19

**Authors:** Veerle Marieke Wintraecken, Sophie Vulik, Sabine de Wild, Carmen Dirksen, Linetta B. Koppert, Jolanda de Vries, Marjolein L. Smidt

**Affiliations:** 1 Department of Surgery, Maastricht University Medical Center+, PO Box 5800, 6202 AZ Maastricht, The Netherlands; 2 GROW - School for Oncology and Developmental Biology, PO Box 616, 6200 MD Maastricht, The Netherlands; 3 Department of Clinical Epidemiology and Medical Technology, Maastricht University Medical Centre CAPHRI – Care and Public Health Research Institute, Maastricht, The Netherlands; 4 Department of Surgical Oncology, Erasmus MC Cancer Institute, University Medical Centre Rotterdam, Rotterdam, The Netherlands; 5 Department of Psychology and Health, Medical Psychology, Tilburg University, P.O. Box 90153, 5000 LE Tilburg, The Netherlands

**Keywords:** Quality of life, Personality traits, Breast neoplasm

## Abstract

**Background:**

Quality of life (QoL) is an important patient-reported outcome that has been studied extensively as an endpoint. There is a growing interest in factors that may influence QoL, such as personality. This descriptive systematic review examined the relationship between personality and QoL in women with non-metastatic breast cancer.

**Methods:**

On November 24th, 2020, with a update on March 7th, 2022, PubMed, PsycINFO, CINAHL, Web of Science and Embase were systematically searched for studies that assessed the direct relationship between personality traits and QoL among adult women diagnosed with non-metastatic breast cancer. The National Institutes of Health Study Quality Assessment Tool was used to assess the quality and risk of bias of the included studies. Three reviewers independently extracted data regarding objectives, population, setting, design, method, outcome measurements and key results. The results are descriptively reported.

**Results:**

Twelve studies (6 cohort studies and 6 cross-sectional studies) were included. Three studies were rated as poor, one study was rated as good, and the remaining studies were rated as moderate. There was a small to moderate effect of personality on QoL as correlation coefficients ranged from 0.10 to 0.77, and the explained variance ranged from 4 to 43%. The (strength of the) relationship depended on the personality trait and QoL domain that was measured and was most apparent for the personality traits ‘optimism’ and ‘trait anxiety’ on psychosocial QoL domains. The results for the personality traits (unmitigated) agency, agreeableness, conscientiousness, novelty seeking, and self-efficacy indicated a smaller but statistically significant correlation between these personality traits and QoL.

**Conclusions:**

The results confirm that personality affects QoL in women with non-metastatic breast cancer and thus provides evidence that personality traits are indeed important influential factors of QoL. It is therefore strongly recommended for all future QoL research to measure personality traits and use these variables as predictive factors, as they are needed to accurately interpret QoL. Information regarding personality traits provide physicians and patients with an interpretation of low or deterioration of QoL, which could guide physicians to improve their patients’ health outcomes and subsequently QoL using psycho-oncological support or treatment.

**Supplementary Information:**

The online version contains supplementary material available at 10.1186/s12885-022-09408-4.

## Background

Quality of life (QoL) is an important patient-reported outcome (PRO) in oncology that has been studied extensively as an endpoint in breast cancer patients [1, 2]. There is a growing interest in factors that may influence QoL, such as personality [1–5].

The relationship between personality traits and health-related QoL (HRQOL) in the general population has been systematically reviewed by Huang and colleagues [6]. The overall conclusion stated that personality traits are indeed related to HRQOL. The review included 76 studies that were published up to 2009. The included populations consisted of individuals with various health states (e.g., cancer, chronic conditions), aging, and healthy. An important limitation of this specific review is the absence of quality and risk of bias assessment of the included studies. In combination with the considerable variance in included populations, and as only three of the included studies examined the relationship between personality traits and HRQOL in breast cancer patients, it is unclear if the results also apply to breast cancer patients in general.

The aim of this systematic review was to provide a descriptive overview of evidence from studies that investigated the direct relationship between personality and QoL in women with non-metastatic breast cancer. The results will not only provide a greater and more accurate understanding of the direct relationship between personality and QoL in these patients, but it can also provide physicians and patients with an explanation of a lower QoL.

## Methods

### Registration and Protocol

This study was performed following the Preferred Reporting Items for Systematic Reviews and Meta-Analyses (PRISMA) guidelines for transparent reporting of systematic reviews [7]. Objectives, methods of analysis, and inclusion criteria were specified in advance and documented in a protocol registered in the International Prospective Register of Systematic Reviews (PROSPERO). Registration number: CRD42020215164*.*

### Search strategy

**Fig. 1:**
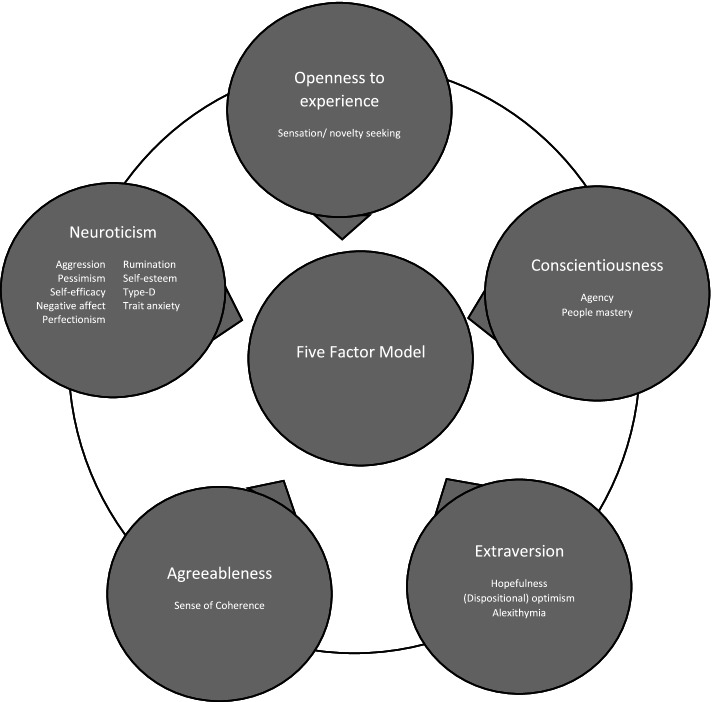
Schematic overview personality dimensions according to the Five Factor Model and the subdivision of single personality traits

In this review the theory of the Five Factor Model (FFM) was used to conceptualize and measure personality and its traits (i.e. aspects of personality that are relatively stable over time and influence behaviour) [8–10]. The FFM measures personality traits at a superordinate level (i.e. five dimensions: neuroticism, extraversion, agreeableness, conscientiousness, and openness to experience) and regard these dimensions as orthogonal (not correlated) [6, 8, 11]. Each dimension comprises six facets, indicating that each domain contains different personality traits [8]. Another way to describe and measure personality is to focus on individual traits rather than personality dimensions. Individual traits have their own specific focus but can also be incorporated into one of the FFM dimensions (see Fig. 1) [6]. On November 24^th^, 2020, PubMed, PsycINFO, CINAHL, Web of Science and Embase were searched, using the keywords personality, QoL, and breast neoplasms (Appendix B provides details regarding the search strategy). These general keywords are most frequently used and led to an extensive search. For all three keywords multiple synonyms were used. To ensure comprehensiveness, individual personality traits were added to the search of personality. This systematic review included observational studies and randomized controlled trials (RCT) to observe the relationship between personality and QoL. RCTs were not included to observe treatment effect, but to capture the above mentioned relationship if measured. Studies were considered eligible if: 1) the studies assessed the direct relationship between personality traits and QoL; 2) study population consisted of female non-metastatic breast cancer patients, ≥ 18 years; 3) personality traits and QoL were assessed with appropriate and validated questionnaires; 4) published in peer-reviewed scientific journals. Due to the heterogeneity in indirect, moderating or mediating effects, it was expected to lead to difficulties when comparing study results or conducting analysis. Therefore, indirect, mediating and moderating effects were excluded.

Studies were excluded if: 1) an indirect relationship, mediating or moderating effect between personality traits and QoL was assessed; 2) published in a language other than English or Dutch. There were no restrictions regarding the time of publication or the length of follow-up. On March 7th, 2022, the search was updated with the same search strategy limiting the time of publication from December 2020 up to January 2022.

### Study selection

Endnote was used as a reference management tool. After deduplication, three reviewers (VW, SV, and SdW) independently screened title and abstract of the retrieved articles using the in- and exclusion criteria, followed by full-text evaluation of potentially eligible studies. Disagreements regarding inclusion were resolved by consensus.

### Data abstraction

The Cochrane data extraction template was used to develop a data extraction sheet. The following data were extracted: objectives, population, setting, design, method, outcome measurements and key results. The data extraction was individually conducted by all reviewers. Disagreements were resolved by consensus. The results are reported using correlation coefficient (r), Odds Ratio (OR) or explained variance (R^2^).

### Risk of bias assessment

The risk of bias was independently assessed by all three reviewers using the Study Quality Assessment Tool from National Institutes of Health (NIH) for observational and cross-sectional studies [12]. Each question was answered with yes (Y), no (N), cannot be determined (CD), not applicable (NA), or not reported (NR). Based on these answers, a final quality rate was given (i.e., poor, fair, or good), as shown in Appendix C. Disagreements were resolved by consensus.

## Results

### Study selection

**Fig. 2:**
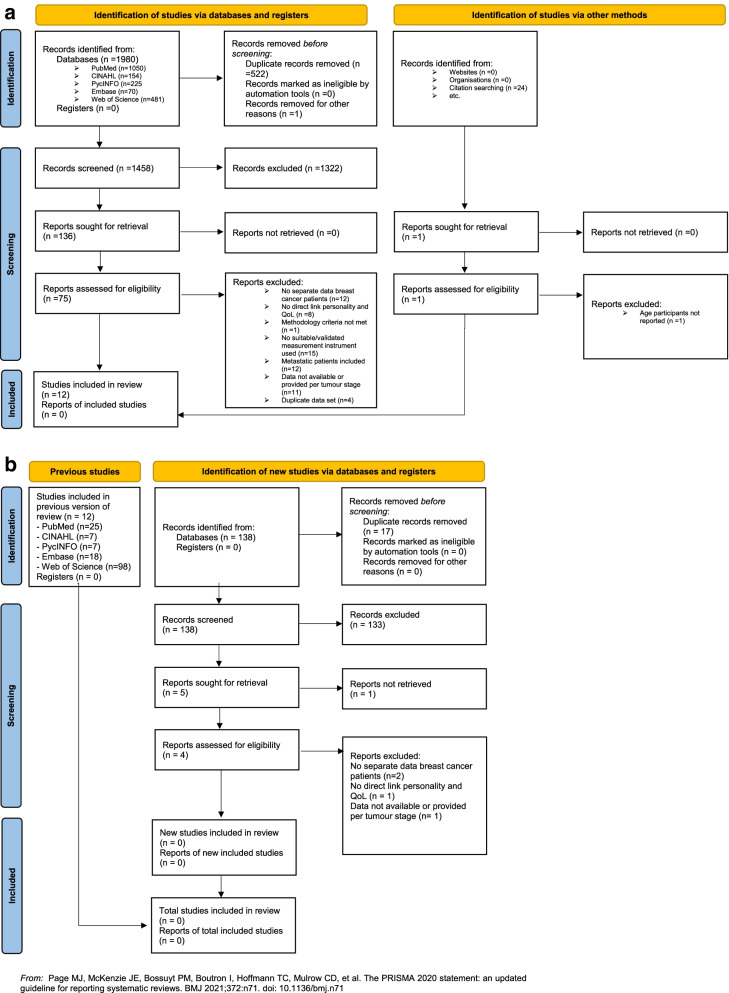
**a** PRISMA 2020 flow diagram for new systematic reviews which included searches of databases, registers and other sources. **b** PRISMA 2020 Flow chart updated search

The database search yielded 1983 articles. Twenty-four records were identified through screening the reference lists of the included studies. After deduplication, 1461 records were screened on title and abstract. Of these, 1386 were excluded. Reasons for ineligibility are detailed in Fig. 2a and b. Of the remaining 75 articles, 63 articles were excluded after full-text screening. Eventually, 12 studies were included in this systematic review (6 cohort studies and 6 cross-sectional studies). Figure 2a and b illustrates the study selection process.

### Risk of bias within studies

The detailed assessment of the risk of bias within the studies using the NIH assessment tool is summarized in appendix C. Three of the included studies were rated as poor, one study was rated as good, and the remaining studies were rated as moderate.

### Study characteristics and results of individual studies

The characteristics and results of individual studies are summarized in Tables 1 and 2, respectively. In the included studies there was heterogeneity in methods, personality trait(s) measured, QoL instruments, and outcomes. Therefore, no statistical method could be used to pool the retrieved data. Results of the included studies are descriptively presented and grouped per personality dimension and the corresponding individual personality traits. Appendix A holds information regarding the definition of each personality trait and the corresponding characteristics and individual personality traits.

**Table 1:** Study characteristics

Author, Year (Country) of study	Study design	Sample size	Personality trait	Personality measure	QoL domain	QoL measure	Statistical analyses	Quality rate
Bellino et al. 2011 (Italy) [13]	CO	57	NS	TCI	PF; RP; BP; GH; VT; SF; IE; MH	SF-36	Univariate regression	Fair
Carver et al. 2006 (USA) [14]	CS	163	O	LOT, LOT-R	NF; PFE; CP; SP; PP; F; SA; BCS	QLACS	Multivariate regression	Fair
Durá-Ferrandis et al. 2016 (USA) [15]	CO	1280	DO	LOT	PF; RF; EF; CF; SF	EORTC QLQ‐C30	Multivariate regression	Fair
Härtl et al. 2010 (Germany) [16]	CO	203	DO; N	FPI-R, LOT	PF; RF; EF; CF; SF	EORTC QLQ‐C30	Multivariate regression	Fair
Petersen et al. 2008 (USA) [17]	CS	268	O; P	MMPI	PH; MH	SF-36, SF-12	T-test and Kruskal–Wallis test	Fair
Popović-Petrović et al. 2018 (Serbia) [18]	CS	64	S	GSES	PWB; SWB; EWB; FWB	FACT-B+4	Hierarchical regression	Poor
Piro et al. 2001 (USA) [19]	CS	74	A; UA	M-EPAQ	EWB; IWB	FACT-B	Hierarchical regression	Poor
Schreier et al. 2004 (USA) [20]	CO	48	TA	STAI	HF; SEC; PS; FA	QLI	Multivariate regression	Fair
Shen et al. 2020 (China) [21]	CS	121	S	GSES	PWB; SWB; EWB; FWB; BCS	FACT-B	Multivariate regression	Fair
van der Steeg et al. 2010 (Netherlands) [1]	CO	222	N; E; OP; AG; C; TA	NEO-FFI, STAI	PH; PSH; LI; SR; EV; SPI	WHOQOL-100	Multivariate regression	Good
Tomich et al. 2006 (USA) [22]	CO	70	O; SE	RSES, LOT	PF; RP; BP; GH; VT; SF; IE; MH	SF-36	Hierarchical regression	Fair
You et al. 2018 (USA) [23]	CS	159	TA	STAI-T	PWB; SWB; EWB; FWB	FACT-B	Hierarchical regression	Poor

Abbreviations: *CO* Prospective Cohort study, *CS* Cross-sectional study

Personality traits

*NS* Novelty Seeking, *O* Optimism, *DO* Dispositional Optimism, *N* Neuroticism, *P* Pessimism, *S* Self-efficacy, *A* Agency, *UA* Unmitigated Agency, *TA *Trait Anxiety, *E* Extraversion, *OP* Openness to Experience, *AG* Agreeableness, *C* Conscientiousness, *SE* Self-esteem

Personality measures

*TCI* The Temperament and Character Inventory, *LOT(-R) *Life Orientation Test(-Revised), *FPI-R *Freiburg Personality Inventory-Revised, *MMPI *Minnesota Multiphasic Personality Inventory, *GSES* General Self-Efficacy Scale, *M-EPAQ* Modified-Extended Personal Attributes Questionnaire, *STAI(-T)* State-Trait Anxiety Inventory(-Trait), *NEO-FFI* NEO Five-Factor Inventory, *RSES* Rosenberg Self-Esteem Scale

QoL domain

*PF* Physical Functioning, *RP* Role function Physical, *BP* Bodily pain, *GH* General Health perceptions, *VT* Vitality, *SF* Social Functioning, *IE* Impact of Emotional problems or daily activities, *MH* Mental health, *NF* Negative feelings, *PFE* Positive Feelings, *CP* Cognitive Problems, *SP* Sexual Problems, *PP* Physical Pain, *F* Fatigue, *SA* Social Avoidance, *BCS* Breast Cancer-specific Concerns, *RF* Role Functioning, *EF* Emotional Functioning, *CF* Cognitive functioning, *PH* Physical Health, *PWB* Physical Well-Being, *SWB *Social/Family Well-Being, *EWB* Emotional Well-Being, *FWB* Functional Well-Being, *HF* Health/Functioning, *SEC* Socioeconomics, *PS* Psychological/Spiritual, *FA *Family, *PSH* Psychological Health, *LI* Level of Independence, *SR* Social Relationships, *EV* Environment, *SPI* Spirituality

QoL measures

*SF-36* Short Form Health Survey-36 items, *SF-12* Short Form Health Survey-12 items, *QLACS* Quality of Life in Adult Cancer Survivors, *EORTC QLQ-C30* European Organization for Research and Treatment for Cancer Quality of Life Questionnaire (version 3), *FACT-B + 4 *Functional Assessment of Cancer Therapy- Lymphedema, *FACT-B* Functional Assessment of Cancer Therapy-Breast, *QLI* Quality of Life Index, *WOQOL-100* World Health Organization Quality of Life assessment instrument

**Table 2:** The relationship between personality traits and QoL. *Note: the included studies by Petersen *et al*., Tomich *et al*., and Härtl *et al*., did not have any specific data and therefore could not be included in the table*

**Personality traits**	**Correlation coefficient (r)**		**Variance in QoL explained by personality traits (%)**		**Odds ratio (CI)**	
**Openness to Experience**						
Novelty seeking [13]			Overall QoL	8%*		
**Conscientiousness** [1]			Overall QoL T3/BCT	9%**		
Agency [19]	EWB	0.25*				
	IWB	0.10				
Unmitigated agency [19]	EWB	-0.21	IWB	35%**		
	IWB	-0.38***				
**Extraversion**						
Optimism [14, 15]	NF	0.36***			EF (AD vs. MHI)	0.43 (0.30–0.65)***
	PFE	0.37***			EF (AD vs. MHI)	0.69 (0.56–0.86)***
	CF	0.15				
	SP	0.36***				
	SA	0.20*				
	F	0.22**				
**Agreeableness **[1]			Overall QoL T3/BCT	4%*		
			Overall QoL T4/BCT	6%*		
**Neuroticism **[1]			Overall QoL T2/MCT +	19%***		
			Overall QoL T3/MTC +	21%***		
			Overall QoL T4/MTC +	20%***		
			Overall QoL T5/MTC +	26%***		
			Overall QoL T5/BCT	34%***		
Self-efficacy [18, 21]	Overall QoL	0.34*—0.49**				
	PWB	0.21—0.39**				
	SWB	0.24—0.27**				
	EWB	0.42**				
	FWB	0.27*—0.35**				
Trait anxiety [1, 20, 23]	Overall QoL	-0.32*—-0.77**	Overall QoL T2/BCT	29%***	Overall QoL	7.81 (2.42–25.72) ***
	PWB	-0.63**	Overall QoL T3/BCT	37%***		
	SWB	-0.50**	Overall QoL T4/BCT	43%***		
	EWB	-0.73**				
	FWB	-0.62**				
	PS	-0.33*				

Abbreviations: *EWB* Emotional Well-Being, *IWB* Interpersonal Well-Being, *NF* Negative Feelings, *PFE* Positive Feelings, *CF* Cognitive functioning, *SP* Sexual problems, *SA* Social Avoidance, *F* Fatigue, *QoL* Quality of Life, *PWB* Physical Well-Being, *SWB* Social/family Well-Being, *FWB* Functional Well-Being, *PS* Psychological/Spiritual, *EF* Emotional functioning, *T2/3/4/5* Time measure point 2/3/4/5, *BCT* Breast-Conserving Therapy, *MTC + * Mastectomy and MTC after BCT, *AD* Accelerated Decline, *MHI* Maintained High, *MD* Moderate Decline

*p < 0.05; **p < 0.01; ***p < 0.001

## Openness to experience

The results from the cohort study by Van der Steeg et al. [1, 4, 25, 26] did not hold evidence that the personality trait openness to experience played a role in predicting patients’ QoL six months post breast cancer diagnosis.

### Novelty seeking

Bellino et al. [13] assessed the effect of novelty seeking (i.e. sensation seeking) on QoL in a cohort study, and showed a clinically meaningful and a statistically significant difference in QoL between baseline and 3 months after surgical intervention (*p* = 0.01) related to novelty seeking (*p* = 0.02). The percentage of variance explained by the relationship between novelty seeking and the change of the QoL scores over time was 8%.

## Conscientiousness

Van der Steeg et al [1, 4, 25, 26] also examined the effect of conscientiousness on QoL. The results show an explained variance of 0.09 (*p* = 0.004), one year post diagnosis.

### Agency

Piro et al. [19] conducted a cross-sectional study and stated that there was a statistically significant correlation between agency and emotional well-being (*r* = 0.25, *p* =  < 0.05), and between unmitigated agency and interpersonal well-being (*r* = -0.38, *p* =  < 0.001). There was no statistically significant correlation between agency and interpersonal well-being, and unmitigated agency with emotional well-being. Agency and unmitigated agency accounted for 35% (34% adjusted) of the variability in interpersonal well-being.

## Extraversion

Van der Steeg et al. [1, 4, 25, 26] also examined the effect of extraversion on QoL. They found no evidence that QoL in breast cancer patients is significantly influenced by the personality trait extraversion.

### Optimism

The effect of optimism on QoL was assessed in three studies. Analyses from a cohort study by Tomich et al., [22] showed no significant association between optimism and QoL for disease-free participants. These findings were confirmed by the results of a hierarchical regression analysis, which revealed that the unstandardized Beta (*B)* of optimism on physical functioning (subscale of QoL) was 1.53 (β 0.14), while the *B* of optimism on mental functioning was 0.97 (β 0.10). None of these findings were statistically significant.

In a cross-sectional study by Carver et al., analysis showed that there was a statistically significant relationship between most QoL domains and optimism, except for the subscales cognitive impairment, pain or financial problems, with correlations ranging between 0.17 and 0.37 (*p*= < 0.001 - < 0.05) [14].

Durá‐Ferrandis et al. [15] performed a cohort study in which they created 3 groups based on QoL scores: 1) consisting of participants beginning with and maintaining near perfect QoL scores over time, 2) consisting of participants with the lowest baseline QoL scores and the steepest rate of decline, and 3) consisting of participants with QoL baseline scores slightly below and only slightly lower declines over time in parallel to group 1. Analysis for emotional functioning showed that the adjusted odds (OR) of being in group 2 (accelerated decline group) was 0.43 less for survivors with higher optimism, compared to group 1 (maintained high group). The OR of being in group 3 (phase shift group) was 0.69 less for survivors with higher optimism compared to group 1. Both ORs appeared to be statistically significant (*p* < 0.001). 

All three studies examining the relationship between optimism and QoL, found that optimistic women scored better on QoL compared to pessimistic women, especially on the QoL domains mental health, emotional functioning, negative feelings, (lack of) positive feelings, and sexual impairment.

## Agreeableness

The explained variance of the personality trait agreeableness on QoL was 0.04 (p 0.037) one year after surgery, and 0.06 (*p* = 0.015), 2 year post diagnosis (van der Steeg et al. [1, 4, 25, 26]).

## Neuroticism

The results from a cohort study by Härtl et al. [16] showed that higher neuroticism scores at baseline predicted a poorer global health status (B -0.25 *p* = 0.001), role functioning (B -0.15 *p* = 0.043), emotional functioning (B -0.18 p 0.015), and cognitive functioning (B -0.16 *p* = 0.013).

Van der Steeg et al. [1, 4, 25, 26] (cohort study) stated that six months after surgery, neuroticism explained up to 26% of the variance in QoL scores in the mastectomy group (*p* < 0.001), and up to 34% of the variance in QoL scores in the lumpectomy group (*p* < 0.001). Irrespective of the type of surgery, high scores on neuroticism were associated with significantly lower overall QoL scores.

### Self-esteem

Tomich et al. [22] also examined the relationship between self-esteem and QoL in their cohort study. The analyses showed no significant relation between self-esteem and physical and mental functioning.

### Self-efficacy

Two studies investigated the relationship between the personality trait self-efficacy and QoL.

A cross-sectional study by Popović-Petrović et al. [18] demonstrated that the *r* was 0.338 (*p* = 0.006) for the total QoL, 0.418 (*p* = 0.001) for emotional well-being, and 0.270 (*p* = 0.031) for functional well-being, indicating significant correlations. When adding self-efficacy as a predictor for QoL in a hierarchical regression analysis, the personality trait self-efficacy was no longer significant.

Results from a cross-sectional study by Shen et al. [21] showed a positive correlation between self-efficacy and the different QoL domains that were all statistically significant, ranging from .493 and .205 (*p* = 0.000 - 0.024). In a multiple stepwise regression model, hope, income, cancer stage, social support and self-efficacy appeared to be a statistically significant indicator for QoL.

To recap, women with high self-efficacy levels assess their QoL higher/better compared to women who do not believe they possess the necessary capabilities.

### Pessimism

Petersen et al. [17] conducted a cross-sectional study and showed that women with pessimistic scores, scored statistical significantly worse on the mental health QoL and Social Support subscale compared to optimistic women. Petersen et al. also assessed the clinical significance which corresponds with previous findings: pessimistic women scored lower on the mental health QoL (52 vs. 47, *p* = 0.0001) but not on the Social Support subscale.

### Trait anxiety

Three studies assessed the effect of trait anxiety on QoL.

According to the results from a cross-sectional study by You et al. [23] Chinese patients had significantly higher trait anxiety levels compared to the US patients. For both the Chinese and the US patients, analyses revealed that there was a significant effect of trait anxiety on QoL, meaning that higher trait anxiety is associated with worse overall QoL (*p* < 0.001). Trait anxiety was associated with all subscales of the FACT-B (physical-, social-, emotional- and functional well-being) with correlation coefficients ranging from 0.50 to 0.77 (all statistically significant, *p* < 0.001).

A cohort study by Van der Steeg et al. [1, 4, 25, 26] demonstrated that at all measured QoL time points, patients with high trait anxiety at baseline had lower QoL scores, which was statistically significant. In this group, up to 43% of the variance in QoL scores was explained by trait anxiety (*p* < 0.001).

In a cohort study by Schreier and Williams [20], results showed that trait anxiety was statistically significant correlated with total QoL (*r* = -0,32, *p * =  < 0.05), and with psychological/spiritual QoL domain (*r* = -0,33, *p* =  < 0.05).

The results show that all included studies examining the relationship between trait anxiety and QoL found a statistically significant correlation between trait anxiety and each of the QoL domains, as well as overall QoL.

### Discussion

This systematic review demonstrates that all, except one, included studies show a small to moderate [27] statistically significant relation between personality traits and overall QoL or a specific QoL domain. All results showed a consistent direction of the relationship between personality traits and QoL. Depending on the personality trait and QoL domain, the correlation coefficients ranged from 0.20 to 0.77, and explained 4% up to 43% of variance in different domains of QoL. Two studies used OR, which varied between 0.43 and 7.81. The results indicate that the association of personality and QoL is most apparent for the personality traits optimism and trait anxiety, and psychosocial QoL domains, such as emotional- or social well-being. These specific associations can be partly explained by the fact that most of the included studies examined the relationship between trait anxiety or optimism and psychosocial QoL domains (5 and 12 studies, respectively). Only five of the included studies reported psychosocial *and *physical QoL scores, of which two found a statistically significant association between the personality traits self-efficacy and trait anxiety, and the QoL domain physical well-being [21, 23]. Based on existing evidence, it was expected that the association between personality traits and QoL domains is the most apparent for psychosocial QoL domains [6, 28, 29].

All included studies in this review examining the effect of trait anxiety on QoL have demonstrated that trait anxiety is negatively related to overall QoL and each QoL domains. This association is confirmed by other research groups [24, 28, 30, 31]. Individuals with high trait anxiety often experience situations as more dangerous or threatening, are more susceptible to stress, and have more state anxiety reactions (a temporary emotional response about a particular situation or activity [32]) than individuals with low trait anxiety [32–35]. Trait anxiety is often seen as part of the personality dimension neuroticism, which is the tendency to experience negative emotions, such as anger and sadness [36, 37]. The results from this review showed that up to 34% of variance in QoL domains can be explained by neuroticism. Individuals with high levels of neuroticism are more prone to stress, high levels of state anxiety, mental and physical health symptoms, and sleep difficulties, which ultimately affects an individual’s short and long term QoL [38–40].

Several studies indicated that the prevalence of anxiety and depression is much lower among optimistic individuals compared to pessimistic individuals [41–44]. This is confirmed by the results of this review, which showed that the association between optimism and several QoL domains is positive, and that higher optimism is related to better QoL (i.e., less negative feelings, sexual problems, social avoidance, and fatigue). Optimistic individuals often have the generalized expectancy that the future holds positive outcomes. Pessimistic individuals have a more negative view on life.

The findings of this review are consistent with existing literature and the 2017 systematic review, which demonstrated that high scores on the personality traits agreeableness, openness to experience, extraversion, conscientiousness and optimism were associated with perception of good health and therefore higher overall QoL, while high level neuroticism was negatively associated with psychological functioning [6, 45–47].

The findings of the current review are also consistent with evidence from diverse groups of non-metastatic and metastatic cancer survivors. Several studies demonstrated that there is a consistent negative association between the personality traits neuroticism and trait anxiety, and QoL for patients with head and neck cancer, gynaecological cancer and colorectal cancer [24, 28, 30, 31]. The association between the personality traits extraversion, dispositional optimism, self-esteem, conscientiousness and QoL is positive [24, 28, 31, 48–52].

Studies examining the relationship between personality traits and QoL in a sample with chronic conditions demonstrated similar results regarding the personality traits conscientiousness, optimism, self-efficacy and neuroticism [53–55]. There was no evidence found for an association between extraversion or agreeableness and QoL.

Based on the abovementioned evidence, high levels of trait anxiety or neuroticism have a negative effect on QoL, irrespective of being diagnosed with cancer, a chronic condition or being a healthy individual. High levels of optimism, self-esteem or self-efficacy have an opposite effect and are associated with better QoL.

## Limitations

The first limitation regards the study quality of the included studies. Three studies were rated as to having poor quality, indicating an increase in the risk of bias (the results of the quality assessment are shown in Appendix C). An important cause of the relative low study quality can be found in the frugal methodological and statistical descriptions. Excluding the results from the studies rated as poor, does not impact the outcome.

The second limitation concerns reporting bias. Most of the included studies did not report non-significant results, which can distort the results from this review. 

The third limitation concerns the lack of information regarding the personality traits of the non-responders in all included studies. Prior studies demonstrated that the personality traits from responders differ significantly from non-responders [56, 57]. However, none of the included studies mentioned if they investigated whether the personality traits of the responders differed from the non-responders.

Another limitation concerns the generalizability of the studies. Several studies did not include relevant demographic information such as comorbidities or response rates, making it difficult to determine whether they had a representative group of breast cancer patients. This could have limited the ability to generalize the results from the study. Moreover, the vast amount of distinct questionnaires or subscales that were used to measure QoL (7 distinct questionnaires) or personality traits (10 distinct questionnaires), limited the ability to compare findings from different studies. Furthermore, we excluded articles that included patients with stage IV breast cancer because there is evidence that stage of disease has a direct effect on QoL [2, 58–60]. However, there are studies reporting that the effect of personality on QoL outweigh the effects of demographic and medical characteristics [1, 13, 61, 62]. This makes it difficult to determine whether the results from this review can be generalizability to a representative group of breast cancer patients including stage IV patients.

Furthermore, personality traits are considered to be a part of someone’s long term personality, which implicates that traits are stable over time. There are however critics of this theory, who believe that experiencing a traumatic event, such as cancer, can alter (to some degree) personality, both negatively as positively [63, 64].

Finally, most of the included studies in this review examined the relationship between trait anxiety or optimism, and QoL. The skewness of included articles that examined these particular relationships, increases the probability of finding significant associations.

The strengths of this current review include the systematic and comprehensive approach to identify studies published up to January 2022, and the quality assessment including reporting biases.

## Clinical implications and recommendations

This review established that there is a statistically relevant relationship between an individual’s personality traits and their QoL, following breast cancer diagnosis. This result validates the use of psychometric tests for all breast cancer patients to provide relevant information for physicians and patients regarding a potential cause of low or deterioration of QoL, and if desired, establish the patient’s need for psycho-oncological support or treatment. The results also imply that measuring QoL without measuring personality traits is of limited value and may lead to inaccurate conclusions regarding QoL scores. All future QoL research should measure personality traits in order to accurately interpret QoL scores.

The strict in- and exclusion criteria that were used in this review, caused a particularly homogeneous group, as opposed to the systematic review conducted in 2017. Nevertheless, when comparing the results from this review with the 2017 review, the conclusions remain the same. This indicates that health state, disease stage or gender, does not affect the relationship between personality traits and QoL.

This review revealed that, although the evidence that personality traits are associated with QoL is strong and consistent, the amount of high- quality QoL studies that measure and stratify for personality traits in their study remains very limited. This review also showed that although there is a substantial variation in QoL and personality traits measurement instruments between studies, the results remain consistent. However, to facilitate the comparison of personality traits between studies, it is recommended to develop a standardized approach to measure these traits. Personality traits should (preferably) be measured as dimensions, to measure a whole range of personality traits along a continuum, to accurately interpret QoL results.

There is strong and consistent evidence that individuals with low levels of optimism, or high level of neuroticism or trait anxiety, are associated with more negative health perceptions, more symptoms, more treatment side effects, and consequently poorer QoL, regardless of their health status, disease stage, or gender [45–47, 65–67]. Characteristics such as age, education, relationship status, and type of surgery are well-established factors influencing QoL. This review provides evidence that personality traits should be added as important influential factors.

## Conclusion

This review has found evidence of a relationship between personality traits and QoL in non-metastatic breast cancer patients, especially for the personality traits ‘trait anxiety’ and ‘optimism’, and psychosocial QoL domains, such as emotional- or social well-being. Personality traits either have a negative or positive relationship, and the strength of the relationship depends on which personality trait and QoL domain(s) are assessed. In order to interpret QoL data accurately, all future QoL research has to stratify for personality traits.

## Supplementary Information

**Additional file 1: Appendix A.** Overview personality traits. **Appendix B.** PubMed Search Strategy. **Appendix C.** Risk of bias assessment. **Appendix D.** PRISMA 2020 checklist. 

## Data Availability

Data sharing is not applicable to this article as no datasets were generated or analysed during the current study.

## Abbreviations

*B*: Unstandardized Beta
*β*: Standardized Beta
FFM: Five Factor Model
HRQOL: Health-Related Quality of Life
NIH: National Institute of Health
OR: Odds Ratio
PRISMA: Preferred Reporting Items for Systematic Reviews and Meta-Analyses
PROs: Patient Reported Outcomes
QoL: Quality of Life
r: Correlation Coefficient
R^2^: Explained variance
