# Supplementary material for: A descriptive systematic review of the relationship between personality traits and quality of life of women with non-metastatic breast cancer
Source: BMC Cancer. 2022 Apr 19;22:426. doi: 10.1186/s12885-022-09408-4 (PMC9020020; doi:10.1186/s12885-022-09408-4)
Supplement: Supplementary file 1 — Additional file 1: Appendix A. Overview personality traits. Appendix B. PubMed Search Strategy. Appendix C. Risk of bias assessment. Appendix D. PRISMA 2020 checklist. [file 12885_2022_9408_MOESM1_ESM.docx]

# Appendix 1

# Overview personality traits

Table 1: Overview personality traits

| **Personality trait** |  | **Characteristics low score** | **Characteristics high score** | **Appropriate and validated measurement instrument** |
| --- | --- | --- | --- | --- |
| **Dimension** | **Individual personality trait**  **(Superordinate factor)** |  |  |  |
| Openness to experience  The open attitude towards other people, beliefs, and experiences. | Sensation seeking/ novelty seeking The need for varied, novel, and complex sensations and experiences and the willingness to take physical and social risks for the sake of such experience. Individuals with high levels of novelty seeking are often more impulsive, extravagant, disorderly, and tend to have higher stress levels. | -Practical  -Conventional  -Prefers routine | -Curious  -Wide range of interest  -Independent | Openness to experience: -NEO Personality Inventory (NEO-PI)^1^ -NEO Five Factor Inventory (NEO-FFI)^2^ -The Revised NEO Personality Inventory (NEO-PI-R)^3^  Sensation/ novelty seeking: -Sensation Seeking Scale (SSS-V)^4,5^ |
| Conscientiousness  One’s orientation towards experiences, goals, and interests of other people. | Agency An individual’s striving to master the environment, to assert the self, to experience competence, achievement, and power  People mastery The feeling as the extent to which a person perceives himself or herself to be in control of events and ongoing situations. | -Impulsive  -Careless  -Disorganized | -Hardworking  -Dependable  -Organized | Conscientiousness: -NEO Personality Inventory (NEO-PI)^1^ -NEO Five Factor Inventory (NEO-FFI)^2^ -The Revised NEO Personality Inventory (NEO-PI-R)^3^  Agency: -Personal Attributes Questionnaire (PAQ)^6,7^ -The Sense of Agency Scale^8,9^  People mastery: -The Pearlin Mastery Scale^10^ -DMQ-18^11^ |
| Extraversion  The degree in which energy, orientation, and attention are focused on the outside world in contrast to the inner world. | Hopefulness A general tendency to construct and respond to the perceived future positively  (Dispositional) optimism A global expectation that good things will be plentiful in the future and bad things will be scarce  Alexithymia Individuals who experience difficulties in identifying and describing their feelings, their cognitive style is concrete and reality-based and they have impoverished inner emotional and fantasy lives. | -Quit  -Reserved  -Withdrawn | -Outgoing  -Warm  -Seeks adventure | Extraversion: -NEO Personality Inventory (NEO-PI)^1^ -NEO Five Factor Inventory (NEO-FFI)^2^ -The Revised NEO Personality Inventory (NEO-PI-R)^3^ -Eysenck Personality Inventory (EPI)^12^ -Eysenck Personality Questionnaire (EPQ)^13^  Hopefulness -Hunter Opinions and Personal Expectations Scale (HOPES)^14^  Dispositional optimism -Life Orientation Test (LOT)^15^ -Attributional Style Questionnaire (ASQ)^16^  Alexithymia -Toronto Alexithymia Scale (TAS)^17-19^ |
| Agreeableness  One’s orientation towards experiences, goals, and interests of other people. | Sense of coherence The extent to which one has a pervasive and enduring, though dynamic, feeling of confidence that (1) the stimuli derived from one’s internal and external environments in the course of living are structured, predictable, and explicable (comprehensibility); (2) the resources are available to one to meet the demands posed by these stimuli (manageability); and (3) these demands are challenges that are worthy of investment and engagement (meaning) | -Critical  -Uncooperative  -Suspicious | -Helpful  -Trusting  -Empathetic | Agreeableness: -NEO Personality Inventory (NEO-PI)^1^ -NEO Five Factor Inventory (NEO-FFI)^2^ -The Revised NEO Personality Inventory (NEO-PI-R)^3^  Sense of coherence -The Sense of Coherence (SOC) Scale^20,21^ |
| Neuroticism  Weighing emotional instability against emotional stability, i.e., the tendency to experience distressing emotions, unrealistic ideas, excessive cravings or urges, and maladaptive coping responses | Aggression An intentional attempt to harm another person  Negative affect Common variance between anxiety, sadness, fear, anger, guilt and shame, irritability, and other unpleasant emotions  Perfectionism Demanding of oneself or others a higher quality of performance than is required by the situation  Pessimism Dimension of generalized expectancies about the occurrence of bad outcomes is one’s future. Pessimism is related to more intense negative feelings such as anxiety, sadness, or despair. Pessimism is associated with health-damaging behaviours.  Rumination An individuals’ self-reflection as well as a repetitive and passive focus on one’s negative emotions  Self-efficacy Beliefs in one’s capabilities to mobilize motivation, cognitive resources and courses of action needed to meet given situational demands. Individuals with high levels of self-efficacy have trust in their own abilities to gain control over events affecting their life, to overcome obstacles, and to perform well.  Self-esteem An individual’s sense of his or her value or worth, or the extent to which a person values, approves of, appreciates, prizes, or likes him or herself. Patients with high levels of self-esteem tend to be more resilient, more likely to engage in healthy behaviours, and better QoL.  Trait anxiety The existence of stable individual differences in the tendence to respond with state anxiety in the anticipation of threatening situations  Type D General propensity to psychological distress that is defined by elevated scores on two broad personality traits, negative affectivity, and social inhibition. | -Calm  -Even-tempered  -Secure | -Anxious  -Unhappy  -Prone to negative emotions | Neuroticism -NEO Personality Inventory (NEO-PI)^1^ -NEO Five Factor Inventory (NEO-FFI)^2^ -The Revised NEO Personality Inventory (NEO-PI-R)^3^ -Eysenck Personality Inventory (EPI)^12^ -Eysenck Personality Questionnaire (EPQ)^13^  -Dutch Personality Inventory (DPI)^22^  Aggression -Buss-Perry Aggression Questionnaire (BPA)^23,24^  Negative affect -The Positive and Negative Affect Schedule (PANAS)^25,26^ - The short form of Eysenck Personality Inventory Emotional Stability Scale (EPI-Q)^27^  Perfectionism -Perfectionism Inventory (PI)^28^ -Frost Multidimensional Perfectionism Scale (FMPS)^29^  Pessimism -Life Orientation Test (LOT)^15^  Rumination -Ruminative Response Scale (RRS)^30,31^  Self-efficacy -General Self-Efficacy Scale (GSES)^32,33^  Self-esteem -Rosenberg Self-Esteem Scale (RSE)^34,35^  Trait anxiety -The State- Trait Anxiety Inventory (STAI)^36^  Type D -Type D Scale (DS)-14/16/24^37^ |

References

1. Costa, P. T., & McCrae, R. R. (1985). *The NEO personality inventory*. Odessa, FL: Psychological Assessment Resources.
2. Costa, P. T., & McCrae, R. R. (1989). NEO five-factor inventory (NEO-FFI). *Odessa, FL: Psychological Assessment Resources*, *3*.
3. Costa Jr, P. T., & McCrae, R. R. (2008). *The Revised NEO Personality Inventory (NEO-PI-R)*. Sage Publications, Inc.
4. Zuckerman, Marvin, et al. "Development of a sensation-seeking scale." Journal of Consulting Psychology 28.6 (1964): 477.
5. Zuckerman, M., & Link, K. (1968). Construct validity for the Sensation Seeking Scale. Journal of Consulting and Clinical Psychology, 32, 420-426.
6. Spence, J. T., Helmreich, R., & Stapp, J. (1975). Ratings of self and peers on sex role attributes and their relation to self-esteem and conceptions of masculinity and femininity. *Journal of personality and social psychology*, *32*(1), 29.
7. Hill, S. A., Fekken, G. C., & Bond, S. L. (2000). Factor structure integrity of the Personal Attributes Questionnaire: An English–French comparison. *Canadian Journal of Behavioural Science/Revue canadienne des sciences du comportement*, *32*(4), 234.
8. Diehl, M., Owen, S. K., & Youngblade, L. M. (2004). Agency and communion attributes in adults' spontaneous self-representations. *International journal of behavioral development*, *28*, 1–15. <https://doi.org/10.1080/01650250344000226>
9. Tapal, A., Oren, E., Dar, R., & Eitam, B. (2017). The sense of agency scale: A measure of consciously perceived control over one's mind, body, and the immediate environment. *Frontiers in psychology*, *8*, 1552.
10. Pearlin, L. I., & Schooler, C. (1978). The Structure of Coping. *Journal of Health and Social Behavior*, *19*(1), 2. doi:10.2307/2136319
11. Morgan, G. A., Wang, J., Barrett, K. C., Liao, H. F., Wang, P. J., Huang, S. Y., & Józsa, K. (2019). The Revised Dimensions of Mastery Questionnaire (DMQ 18): A Manual and Forms for Its Use and Scoring.
12. Eysenck, H. J., & Eysenck, S. B. G. (1975). *Manual of the Eysenck Personality Questionnaire (Junior and Adult)*. Kent, UK: Hodder and Stoughton.
13. Eysenck, H. J., & Eysenck, S. B. G. (1984). Eysenck personality questionnaire-revised.
14. Nunn, K. P., Lewin, T. J., Walton, J. M., & Carr, V. J. (1996). The construction and characteristics of an instrument to measure personal hopefulness. Psychological Medicine, 26, 531-545.
15. Scheier, M. F., Carver, C. S., & Bridges, M. W. (1994). Distinguishing optimism from neuroticism (and trait anxiety, self-mastery, and self-esteem): A re-evaluation of the Life Orientation Test. Journal of Personality and Social Psychology, 67, 1063-1078.
16. Peterson, C., Semmel, A., Von Baeyer, C., Abramson, L. Y., Metalsky, G. I., & Seligman, M. E. (1982). The attributional style questionnaire. *Cognitive therapy and research*, *6*(3), 287-299.
17. Taylor, G. J., Bagby, R. M., Ryan, D. P., Parker, J. D., Doody, K. F., & Keefe, P. (1988). Criterion validity of the Toronto Alexithymia Scale. *Psychosomatic Medicine*, *50*(5), 500–509.
18. Bagby, M., Taylor, G. J., & Ryan, D. (1986). Toronto Alexithymia Scale: Relationship with personality and psychopathology measures. *Psychotherapy and Psychosomatics*, *45*(4), 207-215.
19. Vorst, H. C., & Bermond, B. (2001). Validity and reliability of the Bermond–Vorst alexithymia questionnaire. *Personality and individual differences*, *30*(3), 413-434.
20. Antonovsky, A. (1993). The structure and properties of the sense of coherence scale. Social Science and Medicine, 36, 725–733.
21. Erikson, M., & Lindstrøm, B. (2005). Validity of Antonovsky’s sense of coherence scale: Systematic review. Journal of Epidemiology and Community Health, 59, 460–466.
22. Luteyn, F., Starren, J., & Van Dijk, J. (1985). *Manual for the Dutch Personality Questionnaire*. Amsterdam: Swets & Zeitlinger.
23. Bryant, F. B., & Smith, B. D. (2001). Refining the architecture of aggression: A measurement model for the Buss–Perry Aggression Questionnaire. *Journal of Research in Personality*, *35*(2), 138-167.
24. Buss, A. H., & Perry, M. (1992). The aggression questionnaire. *Journal of Personality and Social Psychology*, *63*(3), 452–459.
25. Watson, D., Clark, L. A., & Tellegen, A. (1988). Development and validation of brief measures of positive and negative affect: the PANAS scales. *Journal of personality and social psychology*, *54*(6), 1063.
26. Crawford, J. R., & Henry, J. D. (2004). The Positive and Negative Affect Schedule (PANAS): Construct validity, measurement properties and normative data in a large non‐clinical sample. *British journal of clinical psychology*, *43*(3), 245-265.
27. Francis, L. J., Lewis, C. A., & Ziebertz, H. (2006). The short-form revised Eysenck personality Questionnaire (EPQ-S): A German edition.
28. Hill, R. W., Huelsman, T. J., Furr, R. M., Kibler, J., Vicente, B. B., & Kennedy, C. (2004). A new measure of perfectionism: The Perfectionism Inventory. *Journal of personality assessment*, *82*(1), 80-91.
29. Frost, R. O., Marten, P., Lahart, C., and Rosenblate, R. (1990). The dimensions of perfectionism. *Cogn. Ther. Res.* 14, 449–468. doi: 10.1007/BF01172967
30. Treynor, W., Gonzalez, R., & Nolen-Hoeksema, S. (2003). Rumination reconsidered: A psychometric analysis. *Cognitive Therapy and Research, 27*, 247–259.
31. Treynor, W., Gonzalez, R., & Nolen-Hoeksema, S. (2003). Rumination reconsidered: A psychometric analysis. *Cognitive therapy and research*, *27*(3), 247-259.
32. Schwarzer, R., & Jerusalem, M. (1995). Generalized Self-Efficacy scale. In J. Weinman, S. Wright, & M. Johnston, Measures in health psychology: A user’s portfolio. Causal and control beliefs (pp. 35-37). Windsor, UK: NFER-NELSON.
33. Chen, G., Gully, S. M., & Eden, D. (2001). Validation of a new general self-efficacy scale. *Organizational research methods*, *4*(1), 62-83.
34. Rosenberg, M., (1979). Conceiving the self. New York: Basic Books.
35. Rosenberg, M. (1965). Society and the adolescent self-image. Princeton, NJ: Princeton University Press.
36. Spielberger, C. D., Gorsuch, R. L., Lushene, R. E., Vagg, P. R., & Jacobs, G. A. (1983). *Manual for the State-Trait Anxiety Inventory*. Palo Alto, CA: Consulting Psychologists Press.
37. Denollet, J. (2005). DS14: Standard assessment of negative affectivity, social inhibition, and Type D personality. Psychosomatic Medicine, 67, 89–97

# Appendix 2

# PubMed Search Strategy

Search 1
"Five Factor Personality Model"[tiab] OR "five factor model"[tiab] " Big five personality traits"[tiab] OR "Big five model"[tiab] OR agreeableness[tiab] OR conscientiousness[tiab] OR extraversion[Mesh] OR neuroticism[Mesh] OR "openness to experience"[tiab] OR "Personality"[Mesh] OR personalit*[tiab] OR personality trait*[tiab] OR "NEO Personality Inventory"[tiab] OR "Eysenck’s Three Factor model"[tiab] OR "Three Factor model"[tiab] OR psychoticism[tiab] OR "trait anxiety"[tiab] OR agency[tiab] OR aggression[tiab] OR Aggressiveness[tiab] OR alexithymia[tiab] OR "dispositional optimism"[tiab] OR optimism[tiab] OR hopefulness[tiab] OR "people mastery"[tiab] OR mastery[tiab] OR "negative affect*"[tiab] OR Negativism[tiab] OR "sense of coherence"[tiab] OR "self efficacy"[tiab] OR "self esteem"[tiab] OR "Type D"[tiab] OR "Type D personality"[tiab] OR "Type D behavior"[tiab] OR "novelty seeking"[tiab] OR "sensation seeking"[tiab] OR perfectionism[tiab] OR "rumination, cognitive"[Mesh] OR rumination, cognitive[tiab]

Search 2
"Quality of Life"[Mesh] OR quality of life[tiab] OR "Well being"[tiab] OR "Health related quality of life"[tiab] OR "life quality"[tiab] OR QOL[tiab] OR HRQOL[tiab]

Search 3
"Breast Neoplasm*"[Mesh] OR "breast neoplasm*"[tiab] OR "mammary neoplasm*"[tiab] OR "breast tumor*"[tiab] OR "mammary tumor*"[tiab] OR "breast tumour*"[tiab] OR "mammary tumour*"[tiab] OR "breast cancer"[tiab] OR "mammary cancer"[tiab] OR "breast carcinom*"[tiab] OR "mammary carcinom*"[tiab]

Search 4
("Five Factor Personality Model"[tiab] OR "five factor model"[tiab] " Big five personality traits"[tiab] OR "Big five model"[tiab] OR agreeableness[tiab] OR conscientiousness[tiab] OR extraversion[Mesh] OR neuroticism[Mesh] OR "openness to experience"[tiab] OR "Personality"[Mesh] OR personalit*[tiab] OR personality trait*[tiab] OR "NEO Personality Inventory"[tiab] OR "Eysenck’s Three Factor model"[tiab] OR "Three Factor model"[tiab] OR psychoticism[tiab] OR "trait anxiety"[tiab] OR agency[tiab] OR aggression[tiab] OR Aggressiveness[tiab] OR alexithymia[tiab] OR "dispositional optimism"[tiab] OR optimism[tiab] OR hopefulness[tiab] OR "people mastery"[tiab] OR mastery[tiab] OR "negative affect*"[tiab] OR Negativism[tiab] OR "sense of coherence"[tiab] OR "self efficacy"[tiab] OR "self esteem"[tiab] OR "Type D"[tiab] OR "Type D personality"[tiab] OR "Type D behavior"[tiab] OR "novelty seeking"[tiab] OR "sensation seeking"[tiab] OR perfectionism[tiab] OR "rumination, cognitive"[Mesh] OR rumination, cognitive[tiab]) AND ("Quality of Life"[Mesh] OR quality of life[tiab] OR "Well being"[tiab] OR "Health related quality of life"[tiab] OR "life quality"[tiab] OR QOL[tiab] OR HRQOL[tiab]) AND ("Breast Neoplasm*"[Mesh] OR "breast neoplasm*"[tiab] OR "mammary neoplasm*"[tiab] OR "breast tumor*"[tiab] OR "mammary tumor*"[tiab] OR "breast tumour*"[tiab] OR "mammary tumour*"[tiab] OR "breast cancer"[tiab] OR "mammary cancer"[tiab] OR "breast carcinom*"[tiab] OR "mammary carcinom*"[tiab])

Restrictions
*Population:* Human
*Language:* Dutch and English
*Methodology:* Case reports; classical articles; clinical study; clinical trial; clinical trial phase 1; clinical trial phase 2; clinical trial phase 3; clinical trial phase 4; comparative study; controlled clinical trial; dataset; evaluation study; journal article; multicenter study; observational study; randomized controlled trial.

## Results PubMed search

**Table 1.** Results PubMed search

|  | **Without restrictions** | **With restrictions** |
| --- | --- | --- |
| **Search 1** | 553, 719 | 415, 049 |
| **Search 2** | 408, 226 | 293, 135 |
| **Search 3** | 399, 282 | 280, 586 |
| **Search 4** | 1194 | 1050 |

**Table 2.** Results PubMed search (time of publication from December, 2020, up to January 2022)

|  | **Without restrictions** | **With restrictions** |
| --- | --- | --- |
| **Search 1** | 40,979 | 2,801 |
| **Search 2** | 59,024 | 7,156 |
| **Search 3** | 31,459 | 2,575 |
| **Search 4** | 108 | 25 |

# Appendix C: Risk of bias assessment

**Table 1: Risk of bias assessment**

Quality of included studies was assessed using the National Institutes of Health (NIH) Quality Assessment tool for Observational Cohort and Cross-Sectional Studies.^1^

| **Author** | **Q1** | **Q2** | **Q3** | **Q4** | **Q5** | **Q6** | **Q7** | **Q8** | **Q9** | **Q10** | **Q11** | **Q12** | **Q13** | **Q14** | **Quality rate^a^** |
| --- | --- | --- | --- | --- | --- | --- | --- | --- | --- | --- | --- | --- | --- | --- | --- |
| Bellino et al.^2^ | Y | Y | NR | Y | NR | Y | N | N | Y | N | Y | NA | N | Y | Fair |
| Carver et al.^3^ | Y | Y | NR | N | NR | NA | NA | N | Y | NA | Y | NA | NA | Y | Fair |
| Durá-Ferrandis et al.^4^ | Y | Y | Y | Y | NR | Y | Y | N | Y | N | Y | NA | CD | Y | Fair |
| Härtl et al.^5^ | Y | Y | NR | Y | NR | Y | Y | N | Y | N | Y | NA | NR | Y | Fair |
| Petersen et al.^6^ | Y | Y | NR | Y | Y | NA | NA | Y | Y | NA | Y | NA | NA | N | Fair |
| Popović-Petrović et al.^7^ | Y | Y | NR | Y | NR | NA | NA | N | Y | NA | Y | NA | NA | Y | Poor |
| Piro et al.^8^ | Y | Y | NR | Y | NR | NA | NA | N | Y | NA | Y | NA | NA | Y | Poor |
| Schreier et al.^9^ | Y | Y | NR | Y | NR | Y | Y | N | Y | N | Y | NA | N | N | Fair |
| Shen et al.^10^ | Y | Y | Y | Y | NR | NA | NA | N | Y | NA | Y | NA | NA | Y | Fair |
| van der Steeg et al.^11^ | Y | Y | Y | Y | Y | Y | Y | Y | Y | N | Y | NA | Y | Y | Good |
| Tomich et al.^12^ | Y | Y | NR | N | NR | Y | Y | N | Y | N | Y | NA | Y | N | Fair |
| You et al.^13^ | Y | Y | Y | N | NR | NA | NA | N | CD | NA | CD | NA | NA | Y | Poor |

Abbreviations: CD cannot be determined; NA, not applicable; NR, not reported; N, no; Y, yes.

### Explanation questions

Q1. Was the research question or objective in this paper clearly stated?
Q2. Was the study population clearly specified and defined?
Q3. Was the participation rate of eligible persons at least 50%?
Q4. Were all the subjects selected or recruited from the same or similar populations (including the same time period)? Were inclusion and exclusion criteria for being in the study prespecified and applied uniformly to all participants?
Q5. Was a sample size justification, power description, or variance and effect estimates provided?
Q6. For the analyses in this paper, were the exposure(s) of interest measured prior to the outcome(s) being measured?
Q7. Was the timeframe sufficient so that one could reasonably expect to see an association between exposure and outcome if it existed?
Q8. For exposures that can vary in amount or level, did the study examine different levels of the exposure as related to the outcome (e.g., categories of exposure, or exposure measured as continuous variable)?
Q9. Were the exposure measures (independent variables) clearly defined, valid, reliable, and implemented consistently across all study participants?
Q10. Was the exposure(s) assessed more than once over time?
Q11. Were the outcome measures (dependent variables) clearly defined, valid, reliable, and implemented consistently across all study participants?
Q12. Were the outcome assessors blinded to the exposure status of participants?
Q13. Was loss to follow-up after baseline 20% or less?
Q14. Were key potential confounding variables measured and adjusted statistically for their impact on the relationship between exposure(s) and outcome(s)?

References

1. Zeng X, Zhang Y, Kwong JS, et al. The methodological quality assessment tools for preclinical and clinical studies, systematic review and meta-analysis, and clinical practice guideline: a systematic review. *J Evid Based Med.* 2015;8(1):2–10. doi.org/10.1111/jebm.12141
2. Bellino, S., Fenocchio, M., Zizza, M., Rocca, G., Bogetti, P., & Bogetto, F. Quality of life of patients who undergo breast reconstruction after mastectomy: effects of personality characteristics. Plastic and reconstructive surgery 2011;127(1);10-17.
3. Carver, C. S., Smith, R. G., Petronis, V. M., & Antoni, M. H. Quality of life among long‐term survivors of breast cancer: different types of antecedents predict different classes of outcomes. Psycho‐Oncology: Journal of the Psychological, Social and Behavioral Dimensions of Cancer 2006;15(9);749-758.
4. Durá‐Ferrandis, E., Mandelblatt, J. S., Clapp, J., Luta, G., Faul, L., Kimmick, G., ... & Hurria, A. Personality, coping, and social support as predictors of long‐term quality‐of‐life trajectories in older breast cancer survivors: CALGB protocol 369901 (A lliance). Psycho‐oncology 2017;26(11);1914-1921.
5. Härtl, K., Engel, J., Herschbach, P., Reinecker, H., Sommer, H., & Friese, K. Personality traits and psychosocial stress: quality of life over 2 years following breast cancer diagnosis and psychological impact factors. Psycho‐Oncology: Journal of the Psychological, Social and Behavioral Dimensions of Cancer 2010;19(2);160-169.
6. Petersen, L. R., Clark, M. M., Novotny, P., Kung, S., Sloan, J. A., Patten, C. A., ... & Colligan, R. C. Relationship of optimism–pessimism and health-related quality of life in breast cancer survivors. Journal of Psychosocial Oncology 2008;26(4);15-32.
7. Popović-Petrović, S., Kovač, A., Kovač, N., Tovilović, S., Novakov, I., & Ćulibrk, D. Secondary lymphedema of the arm, the perception of the disease, self-efficacy and depression as determinants of quality of life in patients with breast cancer. Vojnosanitetski pregled 2018;75(10);961-967.
8. Piro, M., Zeldow, P. B., Knight, S. J., Mytko, J. J., & Gradishar, W. J. The relationship between agentic and communal personality traits and psychosocial adjustment to breast cancer. Journal of Clinical Psychology in Medical Settings 2001;8(4);263-271.
9. Schreier, A. M., & Williams, S. A. Anxiety and quality of life of women who received radiation or chemotherapy for breast cancer. In Oncology nursing forum 2004 (Vol. 31, No. 1, pp. 127-130). ONCOLOGY NURSING SOCIETY.
10. Shen, A., Qiang, W., Wang, Y., & Chen, Y. Quality of life among breast cancer survivors with triple negative breast cancer—role of hope, self-efficacy and social support. European Journal of Oncology Nursing 2020;101771.
11. van der Steeg, A. F., De Vries, J., & Roukema, J. A. Anxious personality and breast cancer: possible negative impact on quality of life after breast-conserving therapy. World journal of surgery 2010;34(7);1453-1460.
12. Tomich, P. L., & Helgeson, V. S. Cognitive adaptation theory and breast cancer recurrence: Are there limits?. Journal of consulting and clinical psychology 2006;74(5);980.
13. You, J., Lu, Q., Zvolensky, M. J., Meng, Z., Garcia, K., & Cohen, L. Anxiety-and health-related quality of life among patients with breast cancer: a cross-cultural comparison of China and the United States. Journal of global oncology 2017;4;1-9.

#

# Appendix D: PRISMA 2020 checklist

| **Section and Topic** | **Item #** | **Checklist item** | **Location where item is reported** |
| --- | --- | --- | --- |
| **TITLE** | | |  |
| Title | 1 | Identify the report as a systematic review. | 1 |
| **ABSTRACT** | | |  |
| Abstract | 2 | See the PRISMA 2020 for Abstracts checklist. | 2+3 |
| **INTRODUCTION** | | |  |
| Rationale | 3 | Describe the rationale for the review in the context of existing knowledge. | 3+4 |
| Objectives | 4 | Provide an explicit statement of the objective(s) or question(s) the review addresses. | 3 |
| **METHODS** | | |  |
| Eligibility criteria | 5 | Specify the inclusion and exclusion criteria for the review and how studies were grouped for the syntheses. | 4+5 |
| Information sources | 6 | Specify all databases, registers, websites, organisations, reference lists and other sources searched or consulted to identify studies. Specify the date when each source was last searched or consulted. | 4 |
| Search strategy | 7 | Present the full search strategies for all databases, registers and websites, including any filters and limits used. | 4+5+Appendix B |
| Selection process | 8 | Specify the methods used to decide whether a study met the inclusion criteria of the review, including how many reviewers screened each record and each report retrieved, whether they worked independently, and if applicable, details of automation tools used in the process. | 5 |
| Data collection process | 9 | Specify the methods used to collect data from reports, including how many reviewers collected data from each report, whether they worked independently, any processes for obtaining or confirming data from study investigators, and if applicable, details of automation tools used in the process. | 5 |
| Data items | 10a | List and define all outcomes for which data were sought. Specify whether all results that were compatible with each outcome domain in each study were sought (e.g. for all measures, time points, analyses), and if not, the methods used to decide which results to collect. | 5 |
|  | 10b | List and define all other variables for which data were sought (e.g. participant and intervention characteristics, funding sources). Describe any assumptions made about any missing or unclear information. | 5 |
| Study risk of bias assessment | 11 | Specify the methods used to assess risk of bias in the included studies, including details of the tool(s) used, how many reviewers assessed each study and whether they worked independently, and if applicable, details of automation tools used in the process. | 5 |
| Effect measures | 12 | Specify for each outcome the effect measure(s) (e.g. risk ratio, mean difference) used in the synthesis or presentation of results. | 5 |
| Synthesis methods | 13a | Describe the processes used to decide which studies were eligible for each synthesis (e.g. tabulating the study intervention characteristics and comparing against the planned groups for each synthesis (item #5)). | 5 |
|  | 13b | Describe any methods required to prepare the data for presentation or synthesis, such as handling of missing summary statistics, or data conversions. | n.a. |
|  | 13c | Describe any methods used to tabulate or visually display results of individual studies and syntheses. | Table 1 |
|  | 13d | Describe any methods used to synthesize results and provide a rationale for the choice(s). If meta-analysis was performed, describe the model(s), method(s) to identify the presence and extent of statistical heterogeneity, and software package(s) used. | n.a. |
|  | 13e | Describe any methods used to explore possible causes of heterogeneity among study results (e.g. subgroup analysis, meta-regression). | n.a. |
|  | 13f | Describe any sensitivity analyses conducted to assess robustness of the synthesized results. | n.a. |
| Reporting bias assessment | 14 | Describe any methods used to assess risk of bias due to missing results in a synthesis (arising from reporting biases). | n.a. |
| Certainty assessment | 15 | Describe any methods used to assess certainty (or confidence) in the body of evidence for an outcome. | n.d. |
| **RESULTS** | | |  |
| Study selection | 16a | Describe the results of the search and selection process, from the number of records identified in the search to the number of studies included in the review, ideally using a flow diagram. | 5+6+Figure 2a + figure 2b |
|  | 16b | Cite studies that might appear to meet the inclusion criteria, but which were excluded, and explain why they were excluded. | Figure 2a + figure 2b |
| Study characteristics | 17 | Cite each included study and present its characteristics. | 5-10 + table 1-2 |
| Risk of bias in studies | 18 | Present assessments of risk of bias for each included study. | Appendix C |
| Results of individual studies | 19 | For all outcomes, present, for each study: (a) summary statistics for each group (where appropriate) and (b) an effect estimate and its precision (e.g. confidence/credible interval), ideally using structured tables or plots. | n.a. |
| Results of syntheses | 20a | For each synthesis, briefly summarise the characteristics and risk of bias among contributing studies. | n.a. |
|  | 20b | Present results of all statistical syntheses conducted. If meta-analysis was done, present for each the summary estimate and its precision (e.g. confidence/credible interval) and measures of statistical heterogeneity. If comparing groups, describe the direction of the effect. | n.a. |
|  | 20c | Present results of all investigations of possible causes of heterogeneity among study results. | n.a. |
|  | 20d | Present results of all sensitivity analyses conducted to assess the robustness of the synthesized results. | n.a. |
| Reporting biases | 21 | Present assessments of risk of bias due to missing results (arising from reporting biases) for each synthesis assessed. | n.d. |
| Certainty of evidence | 22 | Present assessments of certainty (or confidence) in the body of evidence for each outcome assessed. | n.d. |
| **DISCUSSION** | | |  |
| Discussion | 23a | Provide a general interpretation of the results in the context of other evidence. | 10-12 |
|  | 23b | Discuss any limitations of the evidence included in the review. | 12+13 |
|  | 23c | Discuss any limitations of the review processes used. | 12+13 |
|  | 23d | Discuss implications of the results for practice, policy, and future research. | 13+14 |
| **OTHER INFORMATION** | | |  |
| Registration and protocol | 24a | Provide registration information for the review, including register name and registration number, or state that the review was not registered. | 3+4 |
|  | 24b | Indicate where the review protocol can be accessed, or state that a protocol was not prepared. | 3+4 |
|  | 24c | Describe and explain any amendments to information provided at registration or in the protocol. | 3+4 |
| Support | 25 | Describe sources of financial or non-financial support for the review, and the role of the funders or sponsors in the review. | 15 |
| Competing interests | 26 | Declare any competing interests of review authors. | 15 |
| Availability of data, code and other materials | 27 | Report which of the following are publicly available and where they can be found: template data collection forms; data extracted from included studies; data used for all analyses; analytic code; any other materials used in the review. | n.d. |

*From:*  Page MJ, McKenzie JE, Bossuyt PM, Boutron I, Hoffmann TC, Mulrow CD, et al. The PRISMA 2020 statement: an updated guideline for reporting systematic reviews. BMJ 2021;372:n71. doi: 10.1136/bmj.n71 For more information, visit: <http://www.prisma-statement.org/>
